# Enhancing the biosynthesis of riboflavin in the recombinant *Escherichia coli* BL21 strain by metabolic engineering

**DOI:** 10.3389/fmicb.2022.1111790

**Published:** 2023-01-16

**Authors:** Bing Fu, Junhui Ying, Qingwei Chen, Qili Zhang, Jiajie Lu, Zhiwen Zhu, Ping Yu

**Affiliations:** ^1^College of Food Science and Biotechnology, Zhejiang Gongshang University, Hangzhou, Zhejiang, China; ^2^College of Forestry Science and Technology, Lishui Vocational and Technical College, Lishui, Zhejiang, China

**Keywords:** metabolic engineering, riboflavin, FMN riboswitch, CRISPR/Cas9, *Escherichia coli* BL21

## Abstract

In this study, to construct the riboflavin-producing strain R1, five key genes, *ribA*, *ribB*, *ribC*, *ribD*, and *ribE*, were cloned and ligated to generate the plasmid pET-AE, which was overexpressed in *Escherichia coli* BL21. The R1 strain accumulated 182.65 ± 9.04 mg/l riboflavin. Subsequently, the R2 strain was constructed by the overexpression of *zwf* harboring the constructed plasmid pAC-Z in the R1 strain. Thus, the level of riboflavin in the R2 strain increased to 319.01 ± 20.65 mg/l (74.66% increase). To further enhance *ribB* transcript levels and riboflavin production, the FMN riboswitch was deleted from *E. coli* BL21 with CRISPR/Cas9 to generate the R3 strain. The R4 strain was constructed by cotransforming pET-AE and pAC-Z into the R3 strain. Compared to those of *E. coli* BL21, the *ribB* transcript levels of R2 and R4 improved 2.78 and 3.05-fold, respectively. The R4 strain accumulated 437.58 ± 14.36 mg/l riboflavin, increasing by 37.17% compared to the R2 strain. These results suggest that the deletion of the FMN riboswitch can improve the transcript level of *ribB* and facilitate riboflavin production. A riboflavin titer of 611.22 ± 11.25 mg/l was achieved under the optimal fermentation conditions. Ultimately, 1574.60 ± 109.32 mg/l riboflavin was produced through fed-batch fermentation with 40 g/l glucose. This study contributes to the industrial production of riboflavin by the recombinant *E. coli* BL21.

## Introduction

Riboflavin (RF), also known as vitamin B_2_, is a vitamin B complex essential to all life forms (Garcia-Angulo, 2017). Most microorganisms and plants can synthesize RF; however, vertebrates must obtain RF from their diets (Vitreschak et al., 2002). RF involves numerous reactions, and its derivatives flavin mononucleotide (FMN) and flavin adenine dinucleotide (FAD) act as coenzymes for many flavoproteins in many metabolic pathways, such as carbohydrate, protein, vitamin, and fat metabolism (Northrop-Clewes and Thurnham, 2012; Saedisomeolia and Ashoori, 2018). In *Escherichia coli*, RF is successively converted to FMN and FAD by bifunctional RF kinase (RFK, EC 2.7.1.26)/FMN adenylyltransferase (FMNAT, EC 2.7.7.2), which is encoded by *ribF*. In rare cases, RF plays a cofactor role (Juarez et al., 2008). In addition, RF can provide protection against cancer, cardiovascular diseases, brain damage caused by ROS-induced damage in Alzheimer’s disease, lipid peroxidation, and reperfusion oxidative injury and maintain immunity (Powers, 2003; Mazur-Bialy and Pochec, 2016; Saedisomeolia and Ashoori, 2018; Zhao et al., 2018). RF is the second vitamin identified (Northrop-Clewes and Thurnham, 2012) and has been used in many fields, such as pharmaceuticals, cosmetics, and human and animal nutrition (Koizumi et al., 2000).

**Figure 1:**
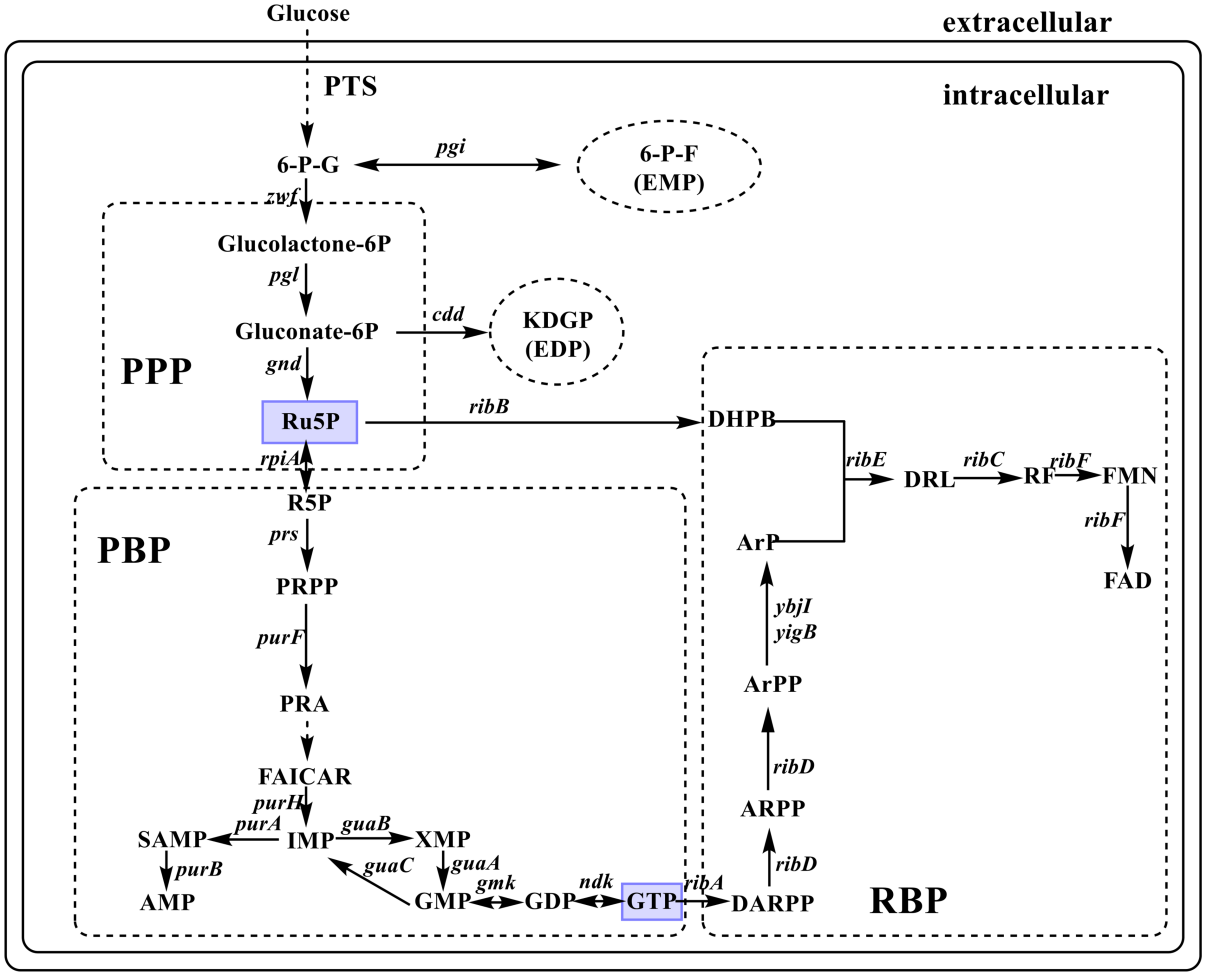
Diagrammatic representation of *de novo* biosynthesis of riboflavin (RF) in *Escherichia coli.* The enzymes encoded by the indicated genes are: *zwf*, glucose-6-phosphate-1-dehydrogenase; *pgi*, phosphoglucose isomerase; *pgl*, 6-phosphogluconolactonase; *edd*, phosphogluconate dehydratase; *gnd*, 6-phosphogluconate dehydrogenase; *rpiA*, ribose-5-phosphate isomerase A; *prs*, ribose-phosphate diphosphokinase; *purF*, amidophosphoribosyltransferase; *purH*, bifunctional AICAR transformylase/IMP cyclohydrolase; *purA*, adenylosuccinate synthase; *purB*, adenylosuccinate lyase; *guaA*, GMP synthase; *guaB*, IMP dehydrogenase; *guaC*, GMP reductase; *gmk*, guanylate kinase; *ndk*, nucleoside diphosphate kinase; *ribA*, GTP cyclohydrolase II; *ribB*, 3,4-dihydroxy-2-butanone-4-phosphate synthase; *ribC,* riboflavin synthase; *ribD*, bifunctional deaminase/reductase; *ribE*, 6,7-dimethyl-8-ribityllumazine synthase; *ybjI* and *yigB*, 5-amino-6-(5-phospho-D-ribitylamino) uracil phosphatase; *ribF*, bifunctional riboflavin kinase/FMN adenylyltransferase. PTS, phosphoenolpyruvate–carbohydrate phosphotransferase system; 6-G-P, D-glucose 6-phosphate; 6-P-F, D-fructose 6-phosphate; KDGP, 2-dehydro-3-deoxy-D-gluconate 6-phosphate; Ru5P, D-ribulose 5-phosphate; R5P, D-ribose 5-phosphate; PRPP, 5-phospho-*α*-D-ribose-1-diphosphate; PRA, 5-phosphoribosylamine; FAICAR, 5-formamido-1-(5-phospho-D-ribosyl)-imidazole-4-carboxamide; IMP, inosine 5′-monophosphate; SAMP, *N^6^*-(1,2-dicarboxyethyl) AMP; AMP, adenosine 5′-monophosphate; XMP, xanthosine 5′-monophosphate; GMP, guanosine 5′-monophosphate; GTP, guanosine 5′-triphosphate; DARPP, 2,5-diamino-6-(5-phospho-D-ribosylamino)pyrimidin-4(3H)-one; ARPP, 5-amino-6-(5′-phosphoribosylamino)uracil; ArPP, 5-amino-6-(5′-phospho-D-ribitylamino)uracil; ArP, 5-amino-6-(1-D-ribitylamino)uracil; DHPB, 3,4-dihydroxy-2-butanone-4-phosphate; DRL, 6,7-dimethyl-8-(1-D-ribityl)lumazine; FMN, flavin mononucleotide; FAD, flavin adenine dinucleotide; EDP, Entner–Doudoroff pathway; EMP, glycolytic pathway; PPP, pentose phosphate pathway; PBP, purine biosynthesis pathway; RBP, riboflavin biosynthetic pathway.

Riboflavin biosynthesis starts with the immediate precursor guanosine 5′-triphosphate (GTP) and D-ribulose 5-phosphate (Ru5P). This RF biosynthetic pathway (RBP) includes seven enzymatic reactions (Figure 1), which are mostly catalyzed by enzymes encoded by *rib* genes, namely, *ribA* (encoding GTP cyclohydrolase II, EC 3.5.4.25), *riB* (encoding 3,4-dihydroxy-2-butanone-4-phosphate synthase, EC 4.1.99.12), *ribC* (encoding RF synthase, EC 2.5.1.9), *ribD* (encoding bifunctional deaminase/reductase, EC 3.5.4.26), and *ribE* (encoding 6,7-dimethyl-8-ribityllumazine synthase, EC 2.5.1.78). However, the enzyme that catalyzes the dephosphorylation of 5-amino-6-(5-phospho-D-ribitylamino) uracil to form 5-amino-6-(1-D-ribitylamino) uracil is unknown. Some evidence has shown that YigB and YbjI in the haloacid dehalogenase superfamily can catalyze this elusive dephosphorylation step (Bacher et al., 2000; Haase et al., 2013; Garcia-Angulo, 2017). *Escherichia coli rib* genes are scattered across the genome, which distinguishes it from the classical RF-producing strain *Bacillus subtilis* with an entire operon (*ribD*-*ribE*-*ribBA*-*ribH*). The coexpression of the key genes of RBP enables *E. coli* to produce RF (Xu et al., 2015).

Enhancing the flux of the purine synthesis pathway by increasing the carbon flux through the pentose phosphate pathway (PPP) and regulating the supply of precursor metabolites can effectively increase RF production (Xu et al., 2015; Liu et al., 2016). The overexpression of *zwf* (encoding glucose-6-phosphate dehydrogenase, EC 1.1.1.49) can increase carbon flow through PPP (Schwechheimer et al., 2016; Sundara Sekar et al., 2017) to enhance the production of RF (Wang et al., 2011; Lu et al., 2012; Lin et al., 2014). A high rate of glucose utilization is important for production cost reduction.

The overexpression of key genes can enhance the yield of a target product, but it inevitably increases metabolic burden and may be counterproductive. Hence, introducing various types of genetic modification through genome engineering, including gene disruption, gene insertion, and point mutation, is essential to the enhancement of pathway efficiency and product yield (Li et al., 2015). CRISPR/Cas systems, comprising clustered regularly interspaced short palindromic repeats (CRISPR) and associated proteins, are innate immune systems in bacteria and archaea (Li et al., 2019; Liu et al., 2020). Bacterial CRISPR/Cas9, which belongs to the type-II CRISPR/Cas system, is widely used in genomic editing in various prokaryotes and eukaryotes (Jiang et al., 2015). The application of this system for gene editing is widely considered the greatest achievement in molecular biology after polymerase chain reaction (PCR) technology (Ledford, 2015).

**Figure 2:**
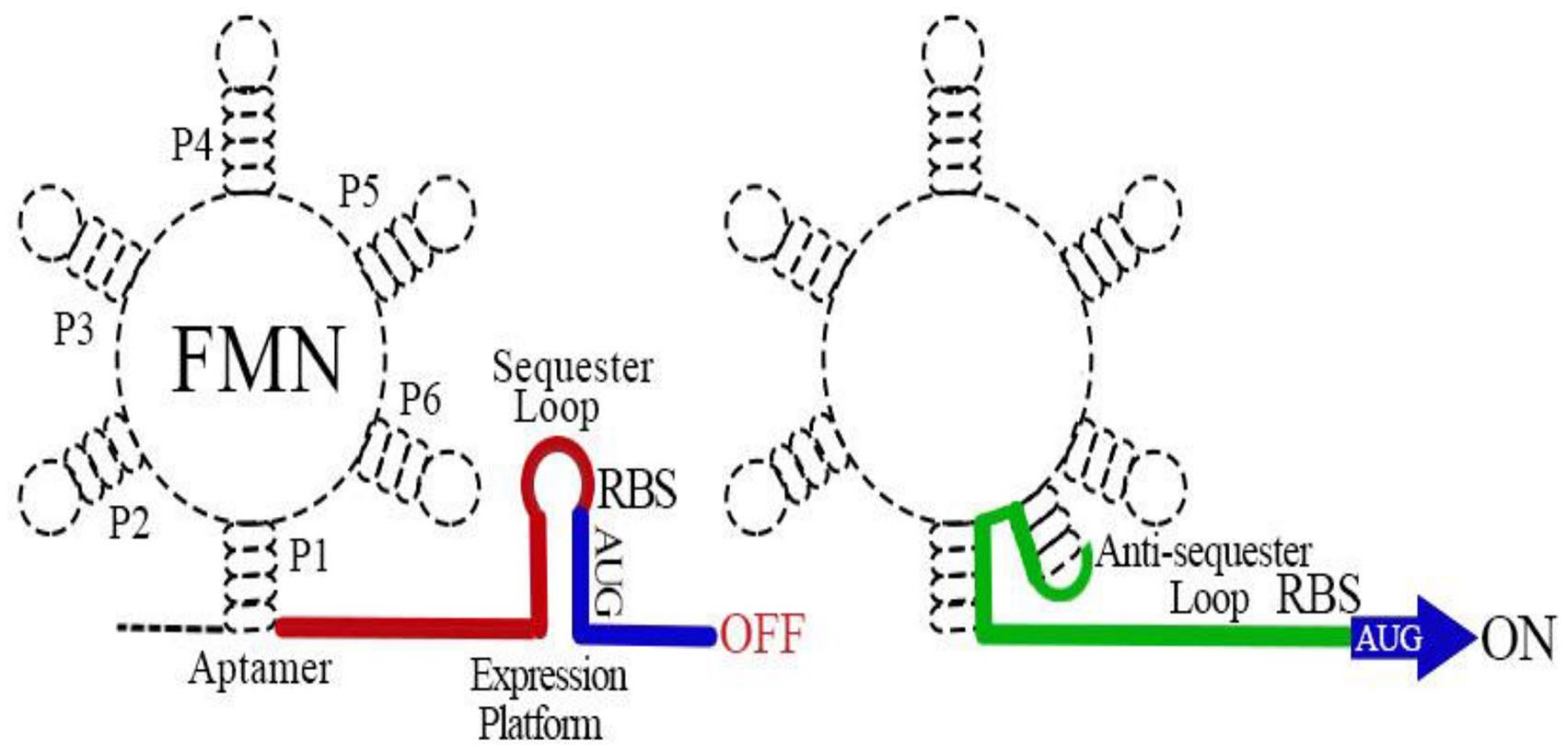
Schematic map of the flavin mononucleotide (FMN) riboswitch mechanism of action. The FMN riboswitch includes two main parts: the 5′ mRNA aptamer and the 3′ downstream expression platform. When FMN binds to the aptamer, the expression platform conforms to a sequester loop (Pritchett and Reddy, 2017) that inhibits *ribB* expression through early termination of transcription of the *ribB* and sequestration of the ribosome binding sequence to prevent translation of fully transcribed *ribB* mRNAs. In the absence of FMN, the aptamer ligand-binding domain adopts an alternative structural conformation that induces an anti-sequester loop (green) in the 3′ downstream expression platform, allowing the continuous expression of *ribB.*

The FMN riboswitch is the most widely distributed regulatory factor of RBP genes (Garcia-Angulo, 2017). It regulates the expression of genes in a *cis* fashion (Howe et al., 2016). In *B. subtilis*, the FMN riboswitch precedes the 5′ UTR *rib* operon mRNA, whereas in *E. coli*, it is present in the 5′ UTR of *ribB* mRNA (Pedrolli et al., 2015a). The FMN riboswitch comprises two distinct functional modules: an aptamer ligand-binding domain responsive to FMN and a downstream expression platform. The regulatory mechanism of the FMN riboswitch is clear. The binding of FMN to an aptamer domain induces a conformational change in the expression platform that regulates *ribB* expression typically through the attenuation of gene expression at the transcriptional and translational levels (Vitreschak et al., 2002; Pedrolli et al., 2015a,b; Howe et al., 2016; Figure 2). The knockout of the FMN riboswitch relieves FMN inhibition, allowing *E. coli* to accumulate RF (Pedrolli et al., 2015a). Owing to the absence of a counterpart in humans, the FMN riboswitch is believed to be a potentially attractive target for antibiotic development (Long et al., 2010; Howe et al., 2016).

In this study, to construct a genetically engineered *E. coli* strain that can produce RF, five key genes in the RBP of *E. coli*, *ribA*, *ribB*, *ribC*, *ribD* and *ribE*, were cloned and ligated to generate the plasmid pET-AE, which was overexpressed in *E. coli* BL21. The *zwf* harboring the constructed plasmid pAC-Z was further expressed to increase the carbon metabolism level and facilitate carbon flow through the PPP. The FMN inhibition of *ribB* was relieved by disrupting the FMN riboswitch in *E. coli* BL21 with CRISPR/Cas9. Through these efforts, the titer of riboflavin was enhanced to 1,574.60 ± 109.32 mg/l through fed-batch fermentation with 40 g/l glucose.

## Materials and methods

### Strains, plasmids, media and reagents

All strains and plasmids used in this study are listed in Table 1. *E. coli* DH5α was used as the host to propagate the plasmid DNA. *E. coli* BL21 was used as an expression host. The compatible plasmids pETDuet-1 and pACYCDuet-1 were used as expression plasmids. Luria-Bertani (LB) medium (10 g/l tryptone, 5 g/L yeast extract, and 10 g/l NaCl) was used for strain culture. M9Y medium (10/L glucose, 6.8 g/l Na_2_HPO_4_, 3.4 g/l KH_2_PO_4_, 0.5 g/l NaCl, 1.0 g/l NH_4_Cl, 5 g/l yeast extract, 2 mM MgSO_4_⋅7H_2_O, and 0.1 mM CaCl_2_; pH 7.2), LBG medium (LB medium with 10 g/l glucose; pH 7.2), and MSY media (10 g/l glucose, 3.8 g/l Na_2_HPO_4_, 1.5 g/l KH_2_PO_4_, 1.0 g/l (NH_4_)_2_SO_4_, 0.2 g/l MgSO_4_⋅7H_2_O, 5 g/l yeast extract, and 2% (*v*/*v*) trace element solution, pH 7.2) were used (Qiu et al., 2004), and the composition of trace element solution was as follows: 100 mg/l ZnSO_4_⋅7H_2_O, 30 mg/l MnCl_2_⋅4H_2_O, 300 mg/l H_3_BO_3_, 200 mg/l CoCl_2_⋅6H_2_O, 10 mg/l CuSO_4_⋅5H_2_O, 20 mg/l NiCl_2_⋅6H_2_O, 30 mg/l NaMoO_4_⋅2H_2_O, and 0.5 M HCl. Ampicillin (100 μg/ml), kanamycin (50 μg/ml), spectinomycin (50 μg/ml), chloramphenicol (25 μg/ml), and isopropylthio-*β*-galactoside (IPTG) were added if necessary. Unless otherwise stated, incubation was carried out at 37°C and 220 rpm, and the IPTG concentration was 1 mM.

**Table 1:** Strains and plasmids used in this study.

Strain/plasmid	Description	Source/reference
**Strain**
*Escherichia coli* DH5α	*Wild-type, F^−^φ80 lac ZΔM15 Δ(lacZYA-arg F) U169 endA1 recA1 hsdR17(rk^−^,mk^+^) supE44λ-thi-1 gyrA96 relA1 phoAe*	TaKaRa
*Escherichia coli* BL21	*Wild-type, F-ompT hsdS_B_(rB-mB^−^) gal dcm (DE3)*	TaKaRa
R1	*E. coli BL21 with* pET-AE	This study
R2	*E. coli BL21 with* pET-AE & pAC-Z	This study
R3	*E. coli BL21 ΔsroG*	This study
R4	*E. coli BL21 ΔsroG with* pET-AE & pAC-Z	This study
**Plasmid**
pETDuet-1	*Expression vector, two MCS, P_T7-LazO_, Amp^R^, compatible with* pACYDuet-1	Novagen
pACYDuet-1	*expression vector, two MCS, P_T7-LazO_, Cm^R^, compatible with* pETDuet-1	Novagen
pET-AE	pETDuet-1 with *ribC*, *ribE*, *ribB*, *ribD*, and *ribA, Amp^R^*	This study
pAC-Z	*pACYCDuet-1 with zwf,Cm^R^*	This study

The FastPure plasmid mini kit, gel DNA extraction mini kit, bacterial DNA isolation mini kit, ClonExpress II one-step cloning kit, and FastPure cell/tissue total RNA isolation kit V2 were purchased from Vazyme Biotech (Nanjing, China). PrimeSTAR HS (premix), a competent cell preparation kit, and restriction enzymes (*Nde*I, *Xho*I, and *Dpn*I) were purchased from Takara Biotech (Dalian, China). Primer synthesis and sequencing were performed by Shanghai Biotech (Shanghai, China).

### Construction and transformation of the expression plasmids

**Figure 3:**
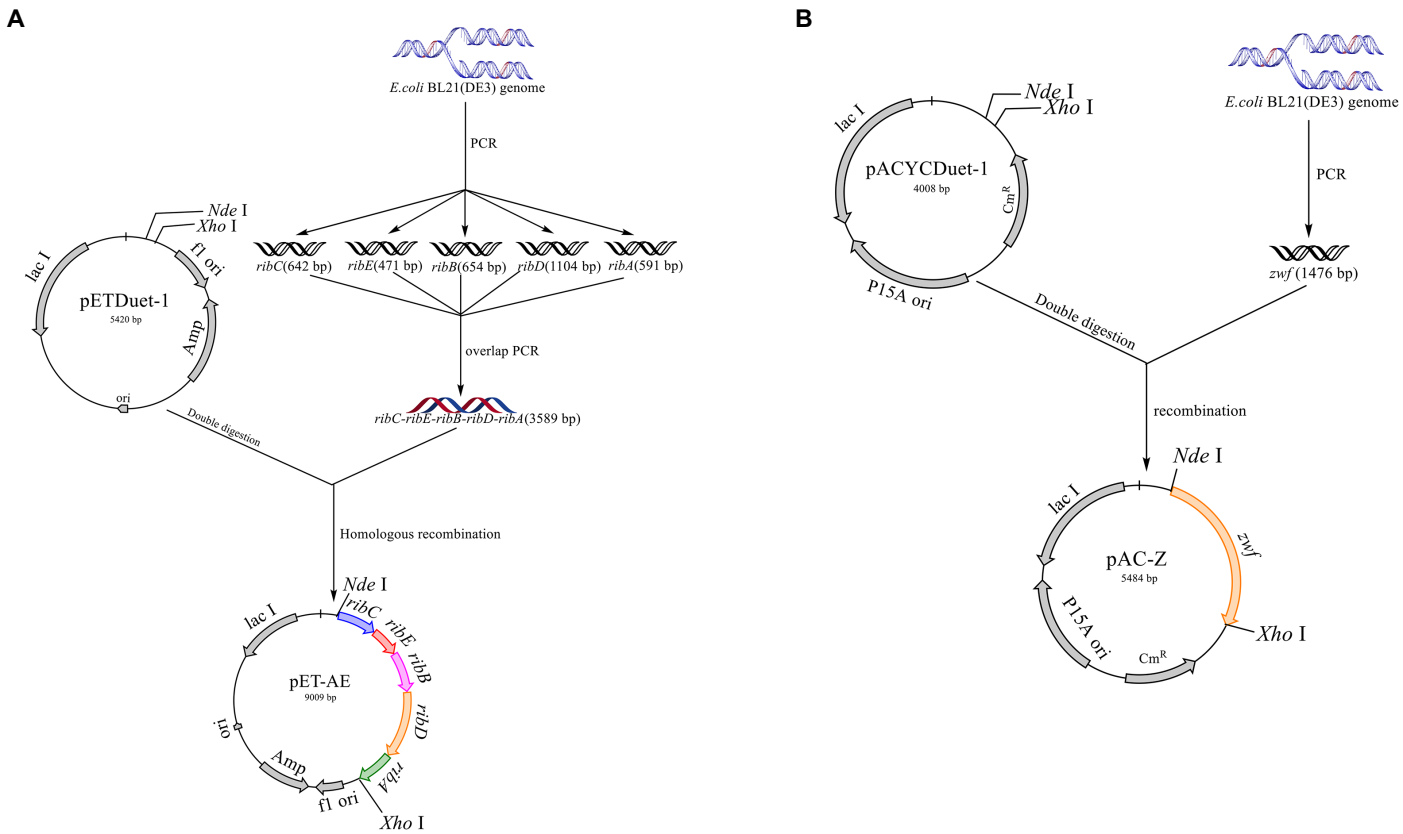
Schematic map of the construction of the plasmids pET-AE and pAC-Z. **(A)** Construction of the plasmid pET-AE; **(B)** the construction of the plasmid pAC-Z.

All primers used in this study are listed in Table 2. The primers *ribA*-F/R, *ribB*-F/R, *ribC*-F/R, *ribD*-F/R, and *ribE*-F/R were used to obtain the gene fragments *ribA*, *ribB*, *ribC*, *ribD*, and *ribE* with the *E. coli* BL21 genome as the template. The artificial operon *ribC*–*ribE*–*ribB*–*ribD*–*ribA* was obtained by ligating the gel extraction products of the five genes through overlap-PCR with the primers *ribC*-F and *ribA*-R. After the plasmid pETDuet-1 was digested by *Nde*I and *Xho*I, the plasmid pET-AE was constructed by ligating the artificial operon *ribC*–*ribE*–*ribB*–*ribD*–*ribA* with the digested plasmid pETDuet-1 using a ClonExpress II one-step cloning kit (Figure 3A). The plasmid pAC-Z was obtained by amplifying the *zwf* gene from the *E. coli* BL21 genome with the primer *zwf*-F/R and conducting homologous combination with pACYCDuet-1, which was digested with *Nde*I and *Xho*I (Figure 3B).

**Table 2:** Primers used in this study.

Primers	Sequences (5′–3′)
*ribA*-F	TGCATTTAGTGGGTGCATAATTAAGAAGGAGATATACCATGCAGCTTAAACGTG
*ribA*-R	GGTTTCTTTACCAGACTCGAGTTATTTGTTCAGCAAATGGCC
*ribB*-F	GAAAGCCATCAAGGCCTAATTAAGAAGGAGATATACCATGAATCAGACGCTACTTTCC
*ribB*-R	GTAATACTCGTCCTGCATGGTATATCTCCTTCTTAATTAGCTGGCTTTACGC
*ribC*-F	AAGTATAAGAAGGAGATATACATATGTTTACGGGGATTGTACAGGGCACCG
*ribC*-R	GCTTCAATAATGTTCATGGTATATCTCCTTCTTAATTAGGCTTCTGTGCCTGG
*ribD*-F	AGCGTAAAGCCAGCTAATTAAGAAGGAGATATACCATGCAGGACGAGTATTAC
*ribD*-R	CACGTTTAAGCTGCATGGTATATCTCCTTCTTAATTATGCACCCACTAAATGC
*ribE*-F	CCAGGCACAGAAGCCTAATTAAGAAGGAGATATACCATGAACATTATTGAAGC
*ribE*-R	GGAAAGTAGCGTCTGATTCATGGTATATCTCCTTCTTAATTAGGCCTTGATGGCTTTC
AE-JC-F	ATGTTTACGGGGATTGTACAGGGCACCGC
AE-JC-R	TTATTTGTTCAGCAAATGGCCCATTTTCTCGGCTTTGG
*zwf*-F	AAGAAGGAGATATACATATGGCGGTAACGCAAACAGCCC
*zwf*-R	AGCAGCGGTTTCTTTACCAGACTCGAGTTACTCAAACTCAT
*zwf*-JC-F	ATGGCGGTAACGCAAAC
*zwf*-JC-R	TTACTCAAACTCATTCCAGGAACGAC
*sorG*-sgRNA-F	ATCCGGACTCTAACCGTCGGGTTTTAGAGCTAGAAATAGC
*sorG*-sgRNA-R	CCGACGGTTAGAGTCCGGATACTAGTATTATACCTAGGAC
*sorG*-armL-F	ATACGGTCAGCGGCAGAAAC
*sorG*-armL-R	TTATAGTGAATCCGCTTATTATTTCAGTGAGGTTTTTTTACCATGAATC
*sorG*-armR-F	TAAAAAAACCTCACTGAAATAATAAGCGGATTCACTATAACGCTAA
*sorG*-armR-R	GTTTACAGCGTTGGGGGGAG
*sorG*-JC-F	CTGACGGTTTTGCGCCATCG
*sorG*-JC-R	TGAATGGGATTACGCGATAAGGT
qribF-F	GAAATCCATGGATCAGACGCTACTTTCCTC
qribF-R	AATGGATCCTCAGCTGGCTTTACGCTCATG

The underlined letters indicate the restriction endonuclease digestion site.

The plasmid pET-AE was transformed into *E. coli* BL21 competent cells to generate the R1 strain by the CaCl_2_-heat shock method (Fu et al., 2021). The R2 strain was constructed by introducing the plasmid pAC-Z into the R1 strain to increase the carbon metabolism level and facilitate carbon flow through the PPP.

### Preparation of cytosolic fractions and SDS-PAGE analysis of the target proteins

A single colony was picked from agar plates and incubated in LB media containing appropriate antibiotics until the OD_600_ values reached 0.6. A final concentration of 2 mM IPTG was added to the cultures to induce the expression of the target proteins. After 6 h of incubation, the cells were collected through centrifugation at 12,000 rpm for 10 min. Phosphate buffer (PBS, pH 7.4) was used to resuspend the obtained cells, and ultrasonic treatment was performed by a sonicator (SCIENTZ-II D, China) to release the proteins (5 s sonication at 150 W, 10 s pause). The resultant crushing solution was centrifuged (12000 rpm, 10 min, 4°C). The precipitate was resuspended in PBS buffer (pH 7.4).

Approximately 10 μl of the precipitate and supernatant were subjected to SDS-PAGE analysis performed on a 12% running gel. Coomassie brilliant blue G-250 was used to stain the resolved proteins.

### Deletion of FMN riboswitch and construction of engineered *Escherichia coli* R3 and R4

The CRISPR/Cas9 system is composed of pCas and pTargetF plasmids. Owing to its efficiency and simplicity, it is widely used in genomic editing (Liu et al., 2017; An et al., 2020).

The designed N20 sequence was evaluated with a web-based tool.^1^ sgRNA was obtained through reverse-PCR using primers with modified N20 at the 3′ end and pTargetF as the template. The PCR products were digested by *Dpn*I at 37°C for 1 h to remove the template. The products were recovered and purified for the transformation of *E. coli* DH5α competent cells and incubated on an LB agar plate (50 μg/ml spectinomycin). Positive clones were screened out through sequencing.

pCas was transformed into *E. coli* BL21 competent cells. The positive clone was incubated in LB medium with kanamycin (50 μg/ml) until the OD_600_ reached 0.2. L-arabinose (30 mM) was added to the culture to induce the expression of Gam, Beta, and Exo. The cultures were collected for the preparation of electrocompetent cells after the OD_600_ reached 0.5. Donor DNA as the repair template which was used for homologous recombination was constructed as follows: approximately 500 bp of the upstream and downstream homologous arms were obtained through PCR, and *E. coli* DL21 genome DNA was used as template. The PCR products were ligated to generate donor DNA through overlap-PCR. The concentrations of *sroG*-sgRNA and donor DNA were measured with a microspectrophotometer (Thermo Scientific NanoDrop, United States). Approximately 400 ng of gRNA and 2 μg of donor DNA were added to *E. coli* BL21 electrocompetent cells, which harbored pCas, after electroporation at 2.5 kV in a 2 mm Gene Pulser cuvette (Bio-Rad, United States). The culture was separated on an LB agar plate (50 μg/ml spectinomycin and kanamycin) and incubated at 30°C overnight. Colony PCR and sequencing were carried out to screen the positive clones.

pCas contains a Cas9 protein from *Streptococcus pyogenes* MGAS5005 with its native promoter, the λ-Red gene under the control of the L-arabinose-inducible promoter (*ParaB*), temperature-sensitive replicon repA101ts for self-curing, and a sgRNA containing an N20 sequence targeting the pTarget *pMB1* replicon (sgRNA*-pMB1*) under the control of the IPTG-inducible promoter (*Ptrc*; Jiang et al., 2015). Plasmid curing was performed as follows: the positive clone was incubated in LB medium containing 0.5 mM IPTG and 50 μg/ml spectinomycin and cultured overnight at 30°C. pCas was cured on LB medium overnight at 42°C. When a strain was subjected to further genomic editing, pCas curing was not performed.

The R3 strain was generated by deleting the FMN riboswitch from primitive *E. coli* BL21 with the CRISPR/Cas9 system according to the above method. The R4 strain was constructed by cotransforming pET-AE and pAC-Z into the R3 strain.

### Transcriptional analysis

Given that the binding of FMN to the FMN riboswitch represses *ribB* expression, the effect of the FMN riboswitch deletion on *ribB* transcript levels was detected through quantitative real-time PCR (qPCR). The *E. coli* BL21, R2, R3, and R4 strains were incubated in LB medium at 37°C. When the OD_600_ reached 0.6, 2 mM IPTG was added to the LB medium to induce the transcription of *ribB*. The cells were harvested when O_600_ reached 1.0. Total RNA was extracted from the *E. coli* cells with a bacterial total RNA isolation kit. After cDNA was synthesized with an M5 Super plus qPCR RT kit (Mei5bio, Beijing, China) with the total RNA as the template, qPCR was performed on a LightCycler480 (Roche, Basel, Switzerland) with 2 × RealStar Green Fast Mixture (GenStar, China).

Differences in *ribB* gene transcript levels between the engineered strains and wild-type *E. coli* BL21 were measured in triplicate, normalized to the 16S internal control, and calculated according to the 2^−∆∆CT^ method (Livak and Schmittgen, 2001).

### Optimization of fermentation conditions

Batch shake-flask culture was used to compare the RF production capacities of different strains. The engineered R4 strain was used to optimize the fermentation conditions (medium, glucose concentration, incubation temperature, and IPTG concentration). The R4 strain was inoculated into 10 ml of LB medium and cultured overnight as a preseed culture. Preseed cultures (0.5 ml) were added to 50 ml of LB and cultured to mid-exponential growth phase at 220 rpm as the seed cultures. One percent of each seed culture (*v*/*v*) was transferred to LB (or M9Y or MSY) medium to produce RF. When the OD_600_ reached 0.6, IPTG was added to induce the expression of target genes for producing RF. If necessary, appropriate antibiotics were added to the medium. Time-course analysis of RF production in a 1 L shake flask was also performed according to the above procedure.

### Detection methods

OD_600_ was measured with a PGENERAL New Century T6 spectrophotometer (Beijing, China). The concentration of glucose was measured by the 3,5-dinitrosalicylic acid method (Hu et al., 2008). The concentration of RF was determined by high-performance liquid chromatography (HPLC; Agilent 1,260 Infinity, CA, United States). Bacterial cultures were diluted before centrifugation because RF is poorly soluble under neutral or acidic conditions and form crystals. The supernatants were filtered (0.22 μm inorganic membrane) and analyzed under the following conditions (Pedrolli et al., 2015a): mobile phase, 18% (*v*/*v*) methanol–20 mM formic acid–20 mM ammonium formate (pH 3.7); flow rate, 0.8 ml/min; injection volume, 10 μl; column, ZORBAX SB-C18 (4.6 × 150 mm; Agilent, CA, United States); and RF detection at 445 nm.

### Statistical analysis

All experiments were performed in triplicate. The results are presented as the mean ± SD of three independent experiments. SPSS 17.0 software was used for statistical analysis. Data were graphed using Origin 8.5 software. The plasmid construction map was generated with ChemDraw Professional 2017 software.

## Results

### Coexpression of key enzymes to enhance the production of RF

For the construction of the RF-producing strain R1, the plasmid pET-AE was transformed into *E. coli* BL21 competent cells. The R2 strain with enhanced glucose utilization ability was obtained by transforming the plasmid pAC-Z into the R1 strain.

**Figure 4:**
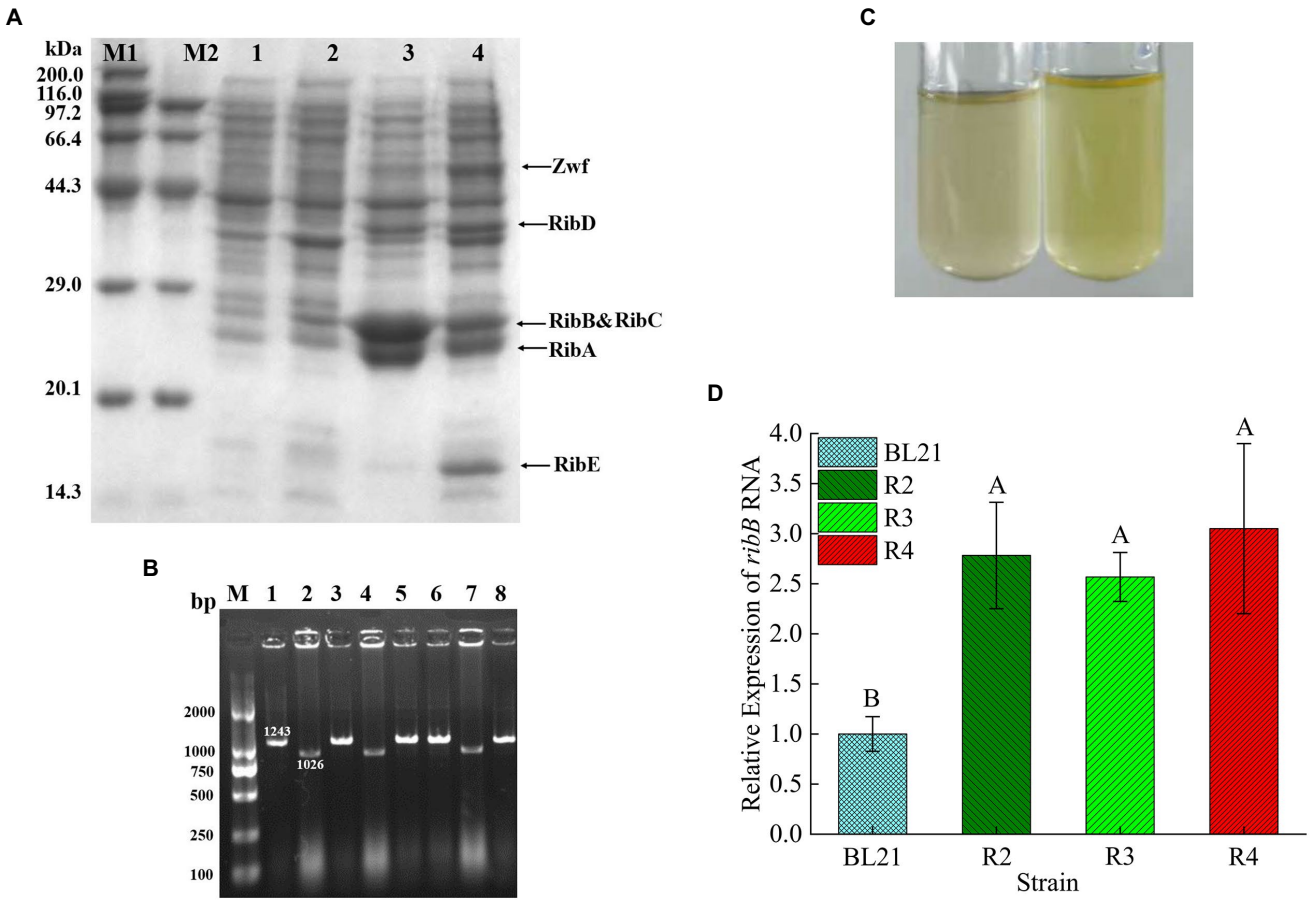
**(A)** SDS-PAGE analysis of the recombinant *E. coli*. Lane M: protein molecular weight marker; Lane 1: precipitated proteins from *E. coli* BL21/pETDuet-1&pACYCDuet-1 after IPTG induction; Lane 2: proteins in supernatant from *E. coli* BL21/pETDuet-1&pACYCDuet-1 after IPTG induction; Lane 3: precipitated proteins from strain R2 after IPTG induction; Lane 4: proteins in supernatant from strain R2 after IPTG induction. **(B)** Colony PCR analysis of *E. coli* BL21▵*sroG*. The length of the positive band was 1,026 bp, while the negative band was 1,243 bp. **(C)** Comparison of the supernatant color after *E. coli* was incubated overnight in 5 ml of LB medium (left: *E. coli* BL21; right: R3). **(D)** Comparison of *ribB* transcript levels of different strains by qPCR. *p* < 0.01. Letters indicate significant differences.

The expression of the target proteins (RibA, RibB, RibC, RibD, RibE, and Zwf) in the recombinant strain R2 was verified through SDS-PAGE. The electrophoresis results are shown in Figure 4A. The apparent molecular weights of the six proteins were 22, 23.4, 23, 40, 16.2, and 52 kDa, respectively, which were consistent with the expected values, indicating that the target proteins were correctly expressed in the recombinant strain R2.

The RF production ability of the recombinant strains was measured through HPLC, and the OD_600_ was measured with a spectrophotometer. The key fermentation parameters (OD_600_ and RF titer) in the strains R1 and R2 are listed in Table 3. After 72 h of incubation, the engineered strain R1 produced 182.65 ± 9.04 mg/l RF, and the OD_600_ reached 7.18 ± 0.36. The engineered strain R2 produced 319.01 ± 20.65 mg/l RF, and the OD_600_ reached 8.76 ± 0.12, showing 74.66 and 22.01% increases compared to the R1 strain, respectively. These results suggested that the overexpression of the key genes in *E. coli* BL21 conferred on the strain the ability to produce RF and that the overexpression of *zwf* on this basis further enhanced RF production and promoted the growth of the recombinant *E. coli* BL21.

**Table 3:** Riboflavin production in various strains.

Strains	OD_600_	Riboflavin titer (mg/L)	Riboflavin yield (mg/g)
*Escherichia coli* BL21	7.13 ± 0.23^b^	0.93 ± 0.31^e^	0.11 ± 0.04^E^
R1	7.18 ± 0.36^b^	182.65 ± 9.04^c^	20.09 ± 1.10^C^
R2	8.76 ± 0.12^a^	319.01 ± 20.65^b^	35.09 ± 2.53^B^
R3	7.02 ± 0.30^b^	4.46 ± 0.23^d^	0.49 ± 0.03^D^
R4	8.29 ± 0.29^a^	437.58 ± 14.36^a^	52.82 ± 2.54^A^

Letters indicate significant differences (*p* < 0.05).

### Deletion of the FMN riboswitch enhances *ribB* transcriptional levels and RF production

To relieve FMN inhibition, a total of 223 bp of nucleotides located upstream of the RBS sequence of *ribB* were deleted. sgRNA was obtained through reverse-PCR using the primer *sroG*-sgRNA-F/R and designated *sroG*-sgRNA. The positive clones were screened out through sequencing after plasmid extraction. The primers *sroG*-armL-F/R and *sroG*-armR-F/R were used to amplify the upstream and downstream homologous arms, *sroG*-Up and *sroG*-Down (with length of approximately 500 bp), with *E. coli* BL21 genome DNA as the template. The primers *sroG*-armL-F and *sroG*-armR-R were used to obtain approximately 1-kb-long donor DNA through overlap-PCR. After cotransformation with *sroG*-sgRNA and donor DNA, the positive clones with 1,026 bp bands were screened out through colony PCR, whereas the negative clones had 1,243 bp bands (Figure 4B). The sequencing results further confirmed the successful knockout of the FMN riboswitch. Before proceeding to the next step, strain R3 was successively subjected to IPTG induction and 42°C culture to cure the *sroG*-sgRNA and pCas. After incubation in 5 ml of LB medium for 24 h, the color of the supernatant turned yellow, indicating that the deletion strain R3 had derepressed the inhibition of RF biosynthesis by FMN compared to the wild-type *E. coli* BL21 (Figure 4C).

Northern blot analysis showed that the deletion of the FMN riboswitch in *E. coli* CmpX13 led to the constitutive synthesis of *ribB* mRNA (Pedrolli et al., 2015a). To compare the effects of FMN riboswitch deletion and *ribB* overexpression on *ribB* transcript levels, the *ribB* transcript levels of *E. coli* BL21, R2, R3, and R4 were quantified through qPCR. As shown in Figure 4D and Table 4, compared to those of *E. coli* BL21, the *ribB* transcript levels of R2, R3, and R4 improved 2.78, 2.57, and 3.05-fold, respectively. These results suggested that the deletion of the FMN riboswitch could derepress the effect of FMN on *ribB* transcription. However, knockout of the FMN riboswitch on the basis of the overexpression of *ribB* resulted in only a slight increase in the transcript level of *ribB* (an increase of 9.7%). This result is consistent with that reported by Pedrolli et al. (2015a).

**Table 4:** *RibB* gene transcript level assays of different strains by qPCR.

Strains	*ribB*	16S	∆Ct	∆∆Ct	2^–ΔΔCt^
*E. coli* BL21	30.10	5.58	24.52	0	1.00
R2	28.66	5.62	23.04	−1.47	2.78
R3	28.62	5.47	23.15	−1.36	2.57
R4	28.47	5.55	22.93	−1.59	3.05

After 72 h of incubation in M9Y medium, the FMN riboswitch deletion strain R3 accumulated 4.46 ± 0.23 mg/l RF, increasing by 3.8-fold compared to the control strain *E. coli* BL21 (0.93 ± 0.31 mg/l). In addition, no significant difference was observed in OD_600_ values between the R3 strain and *E. coli* BL21 (Table 3), suggesting that the deletion of the FMN riboswitch did not affect the growth of the strain. The engineered strain R4 produced 437.58 ± 14.36 mg/l RF, and the OD_600_ value of the cells reached 8.29 ± 0.29. Compared to the engineered strain R2, the RF titer of the engineered strain R4 increased by 0.37-fold (Table 3). This result showed that the deletion of the FMN riboswitch on the basis of the overexpression of key genes was effective in enhancing the RF production capacity of the engineered strain.

### Culture condition optimization and fed-batch fermentation

**Figure 5:**
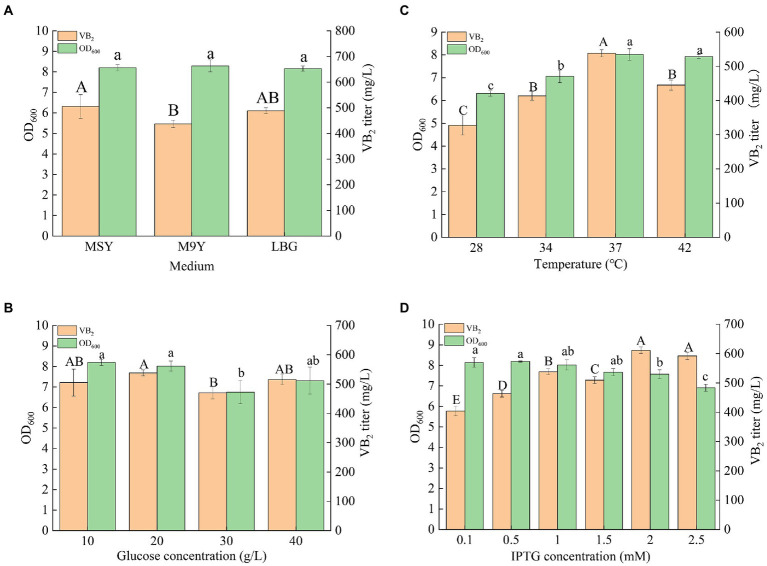
Results of the culture condition optimization of the engineered strain R4: **(A)** fermentation results in different culture media; **(B)** fermentation results with MSY medium containing different concentrations of glucose; **(C)** fermentation results at different temperatures; **(D)** fermentation results at different IPTG concentrations (*p* < 0.05). Letters indicate significant differences.

Riboflavin (RF) production can be further improved by optimizing fermentation conditions, such as culture medium, glucose concentration, culture temperature, and IPTG concentration (Figure 5). The engineered strain R4 was chosen for the optimization of the culture conditions. M9Y, MSY, and LB culture media were used to screen the optimum medium for RF production in this study. As shown in Figure 5A, MSY was the most suitable medium for the fermentation of the R4 strain for RF production, and the titer of RF in MSY medium after incubation for 72 h was 505.01 ± 45.88 mg/l. A variety of metal elements (e.g., Co^2+^, Mn^2+^, etc.) in the trace element solution in MSY may be cofactors for key enzymes in the *de novo* synthesis of RF. Therefore, MSY medium was effective in enhancing RF production in the engineered strain R4. Thus, MSY was selected as the fermentation medium, and a glucose concentration gradient (10, 20, 30, and 40 g/l) was set up to screen the optimal glucose concentration. As shown in Figure 5B, the optimum glucose concentration was 20 g/l, and the titer of RF reached 537.88 ± 10.26 mg/l. A temperature gradient (28, 30, 37, and 42°C) was used to determine the optimum fermentation temperature. MSY containing 20 g/l glucose was used as the fermentation medium. As shown in Figure 5C, the highest RF titer was obtained at 37°C, which was consistent with the optimum growth temperature for *E. coli*. The titer of RF reached 537.88 ± 10.26 mg/l in the engineered strain at 37°C. Since strain R4 contained compatible pET-AE and pAC-Z plasmids under the control of the T7-*Lac* promoter, an induction concentration gradient of IPTG (0.1, 0.25, 0.5, 1.0, 1.5, and 2.0 mM) was set up for the identification of the most suitable IPTG induction concentration. The medium was MSY (20 g/l glucose), and the temperature was 37°C. As shown in Figure 5D, the highest RF titer was obtained at 2 mM IPTG. The RF titer reached 611.22 ± 11.25 mg/l. Taken together, the optimum fermentation conditions of the engineered strain R4 for the production of RF were as follows: medium, MSY; glucose, 20 g/l; temperature, 37°C; and IPTG concentration, 2 mM.

**Figure 6:**
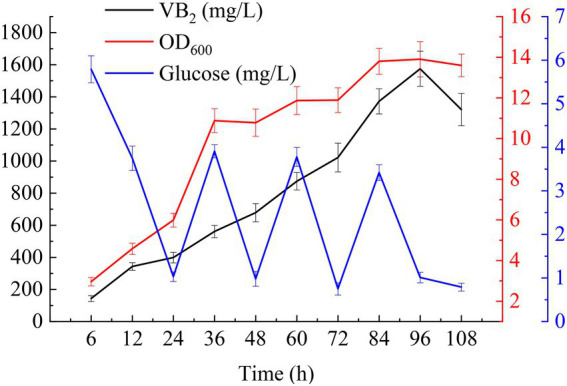
Time-course profiles of key fermentation parameters: glucose concentration, OD_600,_ and RF titer in MSY medium during fed-batch cultivations.

For fed-batch cultivation, 1% (*v*/*v*) seed cultures were added to 250 ml of MSY with 40 g/l glucose in a 1 l shake flask (10 g/l glucose was added to the initial medium and feed medium separately), and the mixture was shaken at 37°C and 220 rpm. 3-(N-morpholino) propanesulfonate (MOPS, 50 mM) was added to the cultures to maintain a neutral pH. IPTG at a final concentration of 2 mM induced the expression of the target genes. Ammonia was used to adjust the pH to 7.2 during fermentation. As shown in Figure 6, with the addition of glucose, the RF titer and the OD_600_ values of the cells increased quickly. The RF titer was the highest after induction with IPTG for 96 h (1574.60 ± 109.32 mg/l with a yield of 12.00 ± 0.83 mg/g glucose). Meanwhile, the OD_600_ value of the cells reached a maximum of 13.9 ± 0.87. Afterward, these values began to decline due to the depletion of glucose and the accumulation of byproducts.

## Discussion

Owing to its wide range of functions in the human body, RF is one of the six main indicators for assessing human body status according to WHO regulations. Given that the human body cannot synthesize RF, humans must consume RF from food, particularly milk, eggs, meat, cheese, and grains. Deficiency in the RF can cause ariboflavinosis (Northrop-Clewes and Thurnham, 2012).

Current strategies for RF production include total chemical synthesis, chemical semisynthesis, and microbial fermentation. Microbial fermentation can be further divided into two types: traditional microbial fermentation and engineered bacterial fermentation. Currently, *B. subtilis* and *Aphis gossypii* are the main strains used in the industrial production of RF (Revuelta et al., 2017). As a common workhorse in fundamental biological research, *E. coli* is used in producing various substances due to its numerous advantages: fast growth rate, clear genetic background, simple genetic manipulation, and low culture requirements. Unlike wild-type *E. coli*, *E. coli* BL21 can accumulate RF under normal culture conditions because of the His115Leu mutation of RibF (Wang et al., 2015). In this study, the titer of RF in the constructed strain R3 was 3.48-fold higher than that in *E. coli* CmpX13 (Pedrolli et al., 2015a). These results demonstrate the potential of *E. coli* BL21 as an RF-producing bacterium.

The overexpression of key genes, the knockout of metabolic branches for the enhancement of the supply of direct precursor substances GTP and Ru5P, deregulation of key enzymes, enhancement of energy generation, reduction of consumption for metabolic maintenance, and optimization of culture media are common strategies for metabolic engineering (Fordjour et al., 2019).

The *de novo* biosynthesis pathway of RF consists of three main pathways: PPP, purine biosynthesis pathway (PBP), and RBP (Figure 1). Metabolic engineering modifications in these pathways are the focus of research, as follows. (1) Metabolic engineering in the PPP phase. The coexpression of the mutant genes *zwf* ^(A243T)^ and *gnd*^(S361F)^ from *Corynebacterium glutamicum* and native *pgl* in *E. coli* MG1655 can enhance RF production (Lin et al., 2014). The coexpression of the mutant genes *zwf*^(A243T)^ and *gnd*^(S361F)^ from *C. glutamicum* in *B. subtilis* also enhances RF production (Wang et al., 2011). Deletion of the *pfkA*, *edd*, and *eda* genes enhances the flux toward PPP (Liu et al., 2016). The knockout of *pgi* can promote metabolic flux toward PPP, which in turn increases RF production (Lin et al., 2014). This can markedly reduce the translation level of the glucose transporter IICB^Glc^ (encoded by *ptsG*) simultaneously (Kimata et al., 2001). Unexpectedly, in this study, *pgi* was disrupted in the engineered strain R4, and the titer of RF decreased by 23% when cultured in M9Y medium without glycine. The disruption of *pgi* decreased the energy supplementation and RF production, and the reduction in OD_600_ verified this effect. (2) Metabolic engineering in the PBP phase. The co-overexpression of *purF*, *purM*, *purN*, *purH*, and *purD*, the key genes in the PBP, increased the ability of *B. subtilis* PK-P to produce RF by 31% (Shi et al., 2009). The purine repressor PurR (encoded by *purR*) tightly regulates the transcription of genes involved in the biosynthesis of inosine 5′-monophosphate (IMP), guanosine 5′-monophosphate (GMP), and adenosine 5′-monophosphate (AMP) from 5-phospho-*α*-D-ribose-1-diphosphate (PRPP; Cho et al., 2011). The deregulation of the purine pathway by disruption of *purR* improves purine nucleotide supply and RF production in *B. subtilis* (Shi et al., 2014). However, in *E. coli*, although the knockout of *purR* increased the expression level of purine synthesis genes, the production of GTP, the direct precursor of RF synthesis, did not increase, similar to the production of RF (Liu et al., 2021). This difference may exist due to the regulation of different genes in the two different microorganisms by PurR. As one of the direct precursors of RF synthesis, GTP is synthesized by PBP. Coexpression of *ndk* and *gmk* can enhance GTP supply and the production of RF in *E. coli* (Xu et al., 2015). PRS and PurF are considered key enzymes in the *de novo* and salvage synthesis of purine nucleotides (Shimaoka et al., 2007; Shi et al., 2009). PRS is inhibited by adenosine diphosphate (ADP), and the *prs*^D128A^ mutation severely affects the affinity of ADP for the PRS binding site (Shimaoka et al., 2007), whereas PurF is inhibited by AMP and GMP. *purF*^(K326Q, P410W, T304E)^ mutations desensitize these inhibitors (Jimenez et al., 2005; Shimaoka et al., 2007). The overexpression of the mutant *prs* and *purF* indeed enhances GTP supplementation, and thus the yield of the target products in different microorganisms (Shimaoka et al., 2007; Xu et al., 2015). Unexpectedly, in this study, the overexpression of mutant *prs* and *purF* did not enhance RF production. The main reason for this may be the excessive metabolic burden. Constitutive expression on chromosomes by gene editing techniques may be a better option. (3) Metabolic engineering in the RBP phase. The coexpression of RBP genes in different microorganisms (*E. coli*, *B. subtilis*, *Pichia pastoris*, and *C. famata*) contributed to the efficient production of RF (Perkins et al., 1999; Marx et al., 2008; Dmytruk et al., 2011; Lin et al., 2014). In the present study, the deletion of the FMN riboswitch derepresses the transcription of *ribB*. Compared to strains that overexpress only *rib* genes and *zwf*, the strain that further knocked out the FMN riboswitch showed a 9.71% increase in *ribB* transcript level but a 37.17% increase in RF production. In *E. coli*, *rib* genes are scattered at different locations on the chromosome, and *ribB* contains an FMN-regulated riboswitch in the upstream 5′ UTR, while no similar regulatory sequences are found in front of other RBP genes, suggesting that *ribB* should be a key gene in RF synthesis (Pedrolli et al., 2015a). These results suggest that RF production can be enhanced by knocking out the FMN riboswitch. To further enhance the titer of RF, reducing the conversion ratio from RF to FMN and FAD is necessary, given that *ribF* is an essential gene for *E. coli* growth (Baba et al., 2006). Thus, ideal options include reducing the expression level of *ribF* or introducing a mutant. Lin et al. (2014) replaced the native RBS of *ribF* with a weaker RBS. This modulation reduced the expression of *ribF* and contributed to RF overproduction. Hu et al. (2021) increased the production of RF by knocking down the *ribF*, *purA*, and *guaC* genes of *E. coli* by using synthetic regulatory small RNA. In *Leuconostoc citreum*, the expression of *ribF* was downregulated by a CRISPR interference system, and production was improved (Son et al., 2020).

The coutilization of carbon sources to reduce production costs has become an important research topic in metabolic engineering (Wu et al., 2016). As the most abundant renewable carbohydrate source on Earth, the efficient use of lignocellulose has attracted the attention of researchers. Although many aspects, such as lignin pretreatment and hydrolysis, are still not well addressed, the coutilization of the hydrolysates of lignocellulose, such as glucose, xylose, and arabinose, has been extensively studied in different organisms (Karhumaa et al., 2006; Jørgensen et al., 2007; Xin et al., 2016). Nevertheless, most industrial microorganisms preferentially utilize glucose over xylose owing to the regulatory phenomenon of carbon catabolite repression (CCR; Gawand et al., 2013). CCR was first studied in microorganisms such as *E. coli*, *B. subtilis*, and *Salmonella typhimurium* during the 1940s, and it emerged as an important gene-regulatory mechanism in bacteria, controlling the expression of approximately 10% of total bacterial genes (Choudhury and Saini, 2017). The accumulation of dephosphorylated EIIA^Glc^ is the main cause of CCR in *E. coli* (Yuan et al., 2020), and the disruption of *ptsG* can relieve CCR, enabling the simultaneous utilization of mixed sugars (Ma et al., 2017; Yuan et al., 2020). In addition to being involved in glucose transport, in some rhizosphere microorganisms (e.g., *B. cereus* C1L), *ptsG* is also involved in root colonization and the production of beneficial metabolites to induce plant systemic disease resistance (Lin et al., 2020). However, the deletion of *ptsG* normally impairs the growth of strains (Chen et al., 2021). In many PTS-strains, this problem is addressed by the coexpression of galactose permease (galP) and glucokinase (glK), which are responsible for glucose transport and phosphorylation (Gawand et al., 2013; Li et al., 2020; Chen et al., 2021). In this study, *ptsG* was disrupted. Although the growth of the bacterium was restored by introducing *galP* and *glK*, the RF production (86.82 ± 3.22 mg/l) was obviously reduced compared to the strain with *pgi* deletion in R4 (165.55 ± 5.89 mg/l) after 24 h incubation. The main reason for this may be that the overexpression of these two genes consumed a considerable amount of energy.

In conclusion, the production of RF was enhanced in the recombinant *E. coli* BL21 strain by combinatorial strategies with key gene overexpression and FMN riboswitch deletion. The overexpression of the key genes enables the *E. coli* BL21 strain to produce a high yield of RF. The knockout of the FMN riboswitch further increases *ribB* transcript levels and RF production. To the best of our knowledge, this is the first use of the knockout of the FMN riboswitch in the construction of an engineered *E. coli* strain for RF production. The engineered strain R4 with the coexpression of five key genes and *zwf* and the deletion of the *FMN riboswitch could accumulate* 611.22 ± 11.25 mg/l RF under optimal fermentation conditions. Ultimately, 1,574.60 ± 109.32 mg/l RF was obtained through fed-batch fermentation with 40 g/l glucose. This study provides a basis for the production of RF in the recombinant *E. coli* BL21 in the future.

## Data availability statement

The original contributions presented in the study are included in the article/supplementary material, further inquiries can be directed to the corresponding author.

## Author contributions

PY conceptualized and designed the experiments. BF, JY, QC, and QZ conducted the experiments. JL and ZZ analyzed the data. PY wrote the manuscript. All authors contributed to the article and approved the submitted version.

## Funding

This study was financed by the Natural Science Foundation of Zhejiang Province, China (No. LY21C200006).

## Conflict of interest

The authors declare that the research was conducted in the absence of any commercial or financial relationships that could be construed as a potential conflict of interest.

## Publisher’s note

All claims expressed in this article are solely those of the authors and do not necessarily represent those of their affiliated organizations, or those of the publisher, the editors and the reviewers. Any product that may be evaluated in this article, or claim that may be made by its manufacturer, is not guaranteed or endorsed by the publisher.

## Footnotes

^1^ http://crispr.tefor.net
